# Leaky Gut As a Danger Signal for Autoimmune Diseases

**DOI:** 10.3389/fimmu.2017.00598

**Published:** 2017-05-23

**Authors:** Qinghui Mu, Jay Kirby, Christopher M. Reilly, Xin M. Luo

**Affiliations:** ^1^Department of Biomedical Sciences and Pathobiology, Virginia-Maryland College of Veterinary Medicine, Virginia Tech, Blacksburg, VA, USA; ^2^Edward Via College of Osteopathic Medicine, Blacksburg, VA, USA

**Keywords:** leaky gut, microbial translocation, gut microbiota, probiotics, autoimmunity

## Abstract

The intestinal epithelial lining, together with factors secreted from it, forms a barrier that separates the host from the environment. In pathologic conditions, the permeability of the epithelial lining may be compromised allowing the passage of toxins, antigens, and bacteria in the lumen to enter the blood stream creating a “leaky gut.” In individuals with a genetic predisposition, a leaky gut may allow environmental factors to enter the body and trigger the initiation and development of autoimmune disease. Growing evidence shows that the gut microbiota is important in supporting the epithelial barrier and therefore plays a key role in the regulation of environmental factors that enter the body. Several recent reports have shown that probiotics can reverse the leaky gut by enhancing the production of tight junction proteins; however, additional and longer term studies are still required. Conversely, pathogenic bacteria that can facilitate a leaky gut and induce autoimmune symptoms can be ameliorated with the use of antibiotic treatment. Therefore, it is hypothesized that modulating the gut microbiota can serve as a potential method for regulating intestinal permeability and may help to alter the course of autoimmune diseases in susceptible individuals.

## Introduction

For digestion and absorption purposes, mammals have developed a very complicated and highly specialized gastrointestinal system maintained by the mucosal barrier (1). However, apart from absorbable nutrients, the intestinal mucosa also faces tremendous exterior antigens, including food antigens, commensal bacteria, pathogens, and toxins. Thus, a specialized barrier function is required to block the entry of diverse exterior antigens while absorbing nutrients. Impressively, in the intestine, the front line of this barrier is maintained by only a single layer of specialized epithelial cells that are linked together by tight junction (TJ) proteins. Many other factors aid in support of this barrier including mucins, antimicrobial molecules, immunoglobulins, and cytokines. If any abnormalities occur among these factors, the intestinal permeability may increase, which is termed a “leaky gut.” A leaky gut allows the entry of exterior antigens from the gut lumen into the host, which may promote both local and systemic immune responses. Multiple diseases may arise or be exacerbated due to a leaky gut, including autoimmune diseases such as inflammatory bowel disease, celiac disease, autoimmune hepatitis, type 1 diabetes (T1D), multiple sclerosis, and systemic lupus erythematosus (SLE) (2–6). Numerous factors can affect gut permeability, such as various diet-derived compounds, alcohol consumption, and gut microbiota dysbiosis. While this review is focused on chronic inflammation and gut barrier functions in mammals, it is worth noting that leaky gut is a phenomenon that is widespread in both mammalian and non-mammalian animals (7). Thus, studies in systems outside of mammals, such as zebrafish (7, 8), can be also helpful in our understanding of the relationship between inflammation and the intestinal barrier.

The gut microbiota has drawn intense attention in the past decade (9). Although scientists have studied gut microbiota for many years, recent advancements in molecular biology including next-generation sequencing technology has enabled researchers to gain new insight in this research field. While we are still far away from clearly understanding the exact roles and effecting modes of gut microbiota, growing evidence suggests that gut microbiota is important in modulating gut permeability and intestinal barrier functions. In this review, we summarize recent advances in the understanding of the leaky gut, bacterial translocation, and gut microbiota dysbiosis, with a particular focus on their association with extraintestinal autoimmune diseases, such as T1D and SLE.

## The Intestinal Barrier

**Figure 1:**
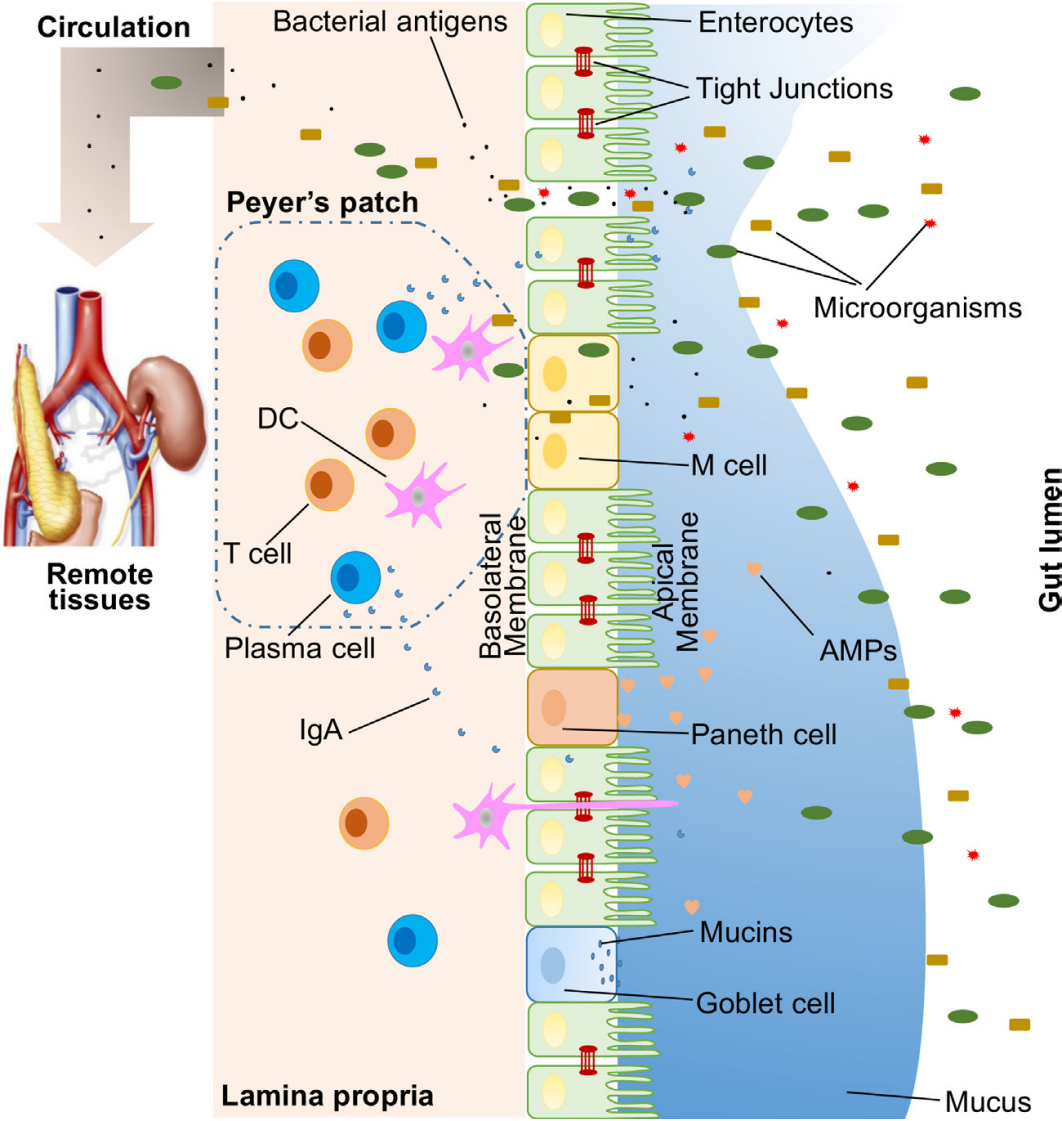
**Illustration of host intestinal barriers, including physical barrier (epithelium, tight junctions, mucus, commensal bacteria), biomedical barrier [antimicrobial proteins (AMPs)], and immunological barrier (lymphocytes and IgA)**. Also shown is the microbial translocation to remote tissues (for example, kidney and pancreas) in the presence of a leaky gut.

A large variety of exogenous substances colonize the gut lumen, such as microorganisms, toxins, and antigens. Without an intact and properly functioning intestinal barrier, these substances can penetrate the tissues beneath the intestinal epithelial lining, diffuse into blood and lymphatic circulations, and disrupt tissue homeostasis. However, there is an efficient multifaceted intestinal barrier system with physical, biochemical, and immunological components that prevents the entry of most pathogens (Figure 1). These components coordinate with each other to prevent uncontrolled translocation of luminal contents into the body. Below is a brief synopsis of the main components comprising the intestinal barrier.

### Physical Barrier

In humans, the intestinal epithelium covers as large as 400 m^2^ of surface area (1). Though only a single layer of cells, the intestinal epithelial cells (IECs) are the mainstay of the intestinal barrier and serve as a physical barrier (Figure 1). There are at least seven types of functional IECs—enterocytes, goblet cells, Paneth cells, microfold cells (M cells), enteroendocrine cells, cup cells, and tuft cells, although the functions of the last two cell populations are not well understood (10). Among all these cell types, enterocytes represent the absolute majority, accounting for at least 90% of crypt cells or villus cells. Enterocytes are absorptive cells and vital for nutrient uptake. However, growing evidence indicates that the functions of enterocytes are not limited to nutrient absorption. For example, enterocytes can control the abundance of Gram-positive bacteria by expressing RegIIIγ, one type of antimicrobial proteins (AMPs) (11–13). All epithelial cell types originate from Lgr5^+^ intestinal epithelial stem cells, which reside within the crypts (14). The turnover rate of IECs is high and the cells are renewed every 3–5 days in the mammalian intestine (10, 15), with the exception being the Paneth cells, which have a life span of about 2 months.

The IEC lining is continuous, and the contact between IECs is sealed by TJs (16). The paracellular pathway, in contrast to transcellular pathway, allows the transport of substances across the gut epithelium through the spaces between IECs. A large variety of molecules, mainly proteins, control the plasticity of TJs. More than 40 TJ proteins have been recognized, including occludin, claudins, junctional adhesion molecule A, and tricellulin (17). Under various pathological conditions, paracellular permeability may be increased, resulting in the entry of unwelcome, potentially harmful molecules.

On top of the gut epithelium, there are two layers of mucus, the inner and outer layers, that cover the whole intestinal epithelial lining and provide physical protection to separate luminal microorganisms from the epithelium. Organized by its major component, a highly glycosylated gel-forming mucin MUC2, the mucus contains diverse molecules including IgA as well as enzymes and proteins, such as lactoferrin (18). Goblet cells are the central cell type for the formation of mucus. They not only produce MUC2 mucin but also secret other mucus components such as ZG16, AGR2, FCGBP, CLCA1, and TFF3 (19, 20). Colitis would spontaneously develop in Muc2-deficient mice, indicating a critical role for MUC2 in mucosal protection (21). In addition to gel-forming mucins, there is another type of mucin that is in close proximity to epithelial cells, called transmembrane mucins. Enterocytes are the main producers of transmembrane mucins (20).

The gut commensal bacteria have been described as one component of the intestinal physical barrier primarily due to its two major functions (22). The first is to promote resistance to the colonization of harmful or pathogenic bacteria species by competing for nutrients, occupying attachment sites, and releasing antimicrobial substances (23, 24). Additionally, the gut microbiota regulates the digestion and absorption of nutrients to supply energy to epithelial cells, which are a major component of the physical barrier (25). A good example of the direct energy supply is the production of short-chain fatty acids by the gut microbiota, which are used by colonocytes for their development and metabolism (26). Taken together, IECs, the mucus layers, and gut microbial residents serve as the physical barrier to limit the entry of unfriendly luminal contents into host tissues.

### Biochemical Barrier

Biochemical molecules with antimicrobial properties exist in the mucus as well as far into the lumen and include bile acids and AMPs (27, 28) (Figure 1). These diverse molecules form a complicated network to reduce the load of colonized bacteria and decrease the chance of contact between luminal antigens and host cells. They are a good supplement to the physical barrier and an essential component of the intestinal barrier function.

The proximal small intestine harbors very few microorganisms (29). But as the distance from the stomach increases, the pH rises and the number of colonized bacteria esculates (30). Facing a large number of microorganisms, which likely outnumber the number of host cells, multiple AMPs are generated to fight against invaders. These AMPs are divided into several types, including α- and β-defensins, C-type lectin, cathelicidin, lysozyme, and intestinal alkaline phosphatase (IAP) (27). Their detailed antimicrobial mechanisms are discussed elsewhere (31). As a major, but not exclusive, producer of AMPs, Paneth cells support and mediate the biochemical barrier function.

### Immunological Barrier

Below the intestinal epithelium, there are organized lymphoid follicles, including the Peyer’s patches and isolated lymphoid follicles. Inside the follicles, a variety of immune cells, including B cells, T cells, dendritic cells (DCs), and neutrophils, orchestrate the immune response by presenting antigens, secreting cytokines, and producing antigen-binding antibodies (Figure 1). In the intestinal epithelium where lymphoid follicles are found, M cells are present that transcytose antigens across the intestinal epithelium to the Peyer’s patches underneath (14). In addition, goblet cells present acquired luminal antigens to CD103^+^ DCs in lamina propria in small intestine by forming goblet cell-associated antigen passages (GAPs) (32, 33). Interestingly, spontaneous antigen presentation was also observed in the colon, but only when the mice were raised germ-free (GF), or housed conventionally but with oral antibiotic treatment (34). This suggests that the antigen uptake process and formation of GAPs are regulated by the colonic microbiota (35). In addition, goblet cells and GAPs are capable of sensing invasive pathogens and inhibiting the translocation of pathogenic bacteria into the host immune system (36). Furthermore, intestinal mononuclear phagocytescan sense and sample luminal contents (37, 38). CX3CR1-expressing cells are responsible for this process, and antigen sampling is dependent on structures called transepithelial dendrites (TEDs) (39, 40). The formation of TEDs is regulated by CX3CR1^+^ macrophages and the expression of CX3CL1 by certain IECs (41, 42).

Another component of the immunological barrier is secretory IgA (SIgA). As the most abundant immunoglobulin in the body, IgA resides primarily on intestinal mucosal surfaces. While some people with selective IgA deficiency appear to be healthy, SIgA is important as it presumably interacts with commensal bacteria to provide protection against pathogens. A unique feature about SIgA is that is structurally resilient in protease-rich environments allowing it to remain functionally active compared to other antibody isotypes on mucosal surfaces (43). In adult humans, about 50 mg/kg of SIgA is produced daily by plasma cells residing in the intestinal lamina propria. Finally, SIgA can be transcytosed through the epithelium and secreted into the gut lumen.

Though not mentioned here, self-modulating factors, such as nerves and diverse cytokines, are also important for maintaining the normal functions of the intestinal barrier.

## Gut Microbiota and the Intestinal Barrier

Microbiota can be sensed by the host through pattern recognition receptors (PRRs), such as toll-like receptors (TLRs) and nucleotide-binding oligomerization domain (NOD)-like receptors (NLRs). In the gut, the bacteria–host communications are largely dependent on the recognition of microbe-associated molecular patterns by PRRs expressed on immune and non-immune cells. Certain microbiota, bacterial products, and metabolites affect the intestinal barrier function and are responsible for the subsequent breakdown of tissue homeostasis. When there is a leaky gut, commensal bacteria in gut lumen, together with their products, are able to escape the lumen of the gut, which may induce inflammation and cause systemic tissue damages if translocated into peripheral circulation (Figure 1). This process of translocation is called microbial translocation (44).

Evidence from GF animals suggests that the development and function of the intestinal barrier are dependent on microbiota. In GF animals, due to the lack of bacterial stimulations, the thickness of the mucus layers is extremely reduced (45–48). The important role of gut microbiota in modulating mucin production from goblet cells is further evidenced in animals with lower loads of bacteria (49, 50). The thinner mucus layers would allow for bacteria penetration, which may initiate inflammation and inflammatory diseases such as colitis (46, 51). Commensal bacteria, or bacterial products such as lipopolysaccharide (LPS) and peptidoglycan, can restore the mucus layers (46, 47). A balance exists between commensal bacteria and the mucus layers, and together they contribute to the maintenance of gut homeostasis (48). Within the mucus layers, there are diverse secreted AMPs that can clear pathogens and control the colonization of commensal bacteria. Reciprocally, the production of some AMPs is regulated by microbiota and/or their products. For instance, RegIIIγ is the AMP necessary for physically separating commensal bacteria from intestinal epithelium (11). RegIIIγ has been shown to be suppressed in alcoholic patients and mice receiving ethanol treatment (52, 53). Prebiotics administration, or increasing probiotic *Lactobacilli* and *Bifidobacteria*, has been shown to restore the properties of RegIIIγ and control bacterial overgrowth (53). Ang4, a member of angiogenin family, is another example where gut commensals are known to modulate AMP production. In one study, Gordon and coworkers found that the production and secretion of Ang4 from mouse Paneth cells were induced by a predominant gut microflora, *Bacteroides thetaiotaomicron* (54). Therefore, the antibacterial activity of Ang4 against microbes in gut lumen is, in turn, dependent on the existence of certain commensal species.

In addition, an interaction exists between gut microbes and AMPs, such as IAP. Predominately produced by IECs, IAP is active either anchored on the epithelium membrane or secreted into gut lumen (55, 56). In IAP-deficient mice, it was noted that there were fewer microbes and an altered bacteria composition compared to control wild-type animals. In particular, the researchers noted a decrease in *Lactobacillaceae* (57, 58). Upregulated IAP activity can selectively increase LPS-suppressing bacteria (e.g., *Bifidobacterium*), while reducing LPS-producing bacteria (e.g., *Escherichia coli*) (59). Having the capacity to inactivate LPS *in vivo*, IAP is vital in preventing the translocation of LPS, the pro-inflammatory stimulus originated from bacteria (60, 61). Of note, the expression of IAP relies on the presence of microbiota. In GF zebrafish, the colonization of commensals, or even supplying LPS alone, could sufficiently induce IAP expression (62). It is worth mentioning that IAP can also regulate TJ proteins to enhance barrier function through increasing ZO-1, ZO-2, and occludin expression (63). Several others have also reported on the various types of AMPs and their function in the microbiota (64, 65).

Intestinal epithelial cells compose the single layer of intestinal epithelium, and the generation of new IECs from local intestinal stem cells is vital in maintaining the barrier function due to the high frequency of apoptosis and shedding of IECs (66). As much as 10% of all the gene transcriptions, especially genes related to immunity, cell proliferation, and metabolism, in IECs are regulated by gut microbiota (67). In GF and antibiotic-treated mice, epithelial proliferation rate is reduced, suggesting the role of microbiota on epithelium cell renewal (68, 69). LPS from *E. coli* can induce cell shedding in a dose-dependent manner (70, 71). Colonization of *Bifidobacterium breve*, or more precisely its surface component, exopolysaccharide, can positively modulate LPS-induced epithelium cell shedding through epithelial MyD88 signaling (70). The renewal of IECs relies on the activity of intestinal stem cells that are located at the base of crypts and express TLR4, the LPS receptor. TLR4 activation has been demonstrated to inhibit proliferation and promote the apoptosis of Lgr5^+^ intestinal stem cells. In mice bearing selective TLR4 deletion in intestinal stem cells, LPS is no longer able to inhibit the renewal of IECs (72). This process was found to be mediated by the p53-upregulated modulator of apoptosis (PUMA) as TLR4 activation in mice lacking PUMA was unaltered. Apart from LPS, bacterial metabolites, particularly butyrate, have also been identified as inhibitors of intestinal stem cell proliferation (73). The intestinal crypt architecture protects the intestinal stem cells from the negative effect of butyrate. As gatekeepers for the paracellular pathway, TJ complexes are also major targets of microbiota regulation (74). This is particularly true for certain probiotic species including, but not limited to, *Lactobacillus rhamnosus* (75–78), *Streptococcus thermophilus* (79), *Lactobacillus reuteri* (80), and *Bifidobacterium infantis* (81).

## Mechanisms of Leaky Gut

A large variety of gut barrier disruptors and/or gut microbiota disturbers may potentially result in microbial translocation and subsequent inflammation locally and systemically. These include diet, infections, alcohol consumption, and burn injury.

### Diet-Induced Gut Leakiness

Nutrients and food ingredients have been reported to contribute to the maintenance or alterations of gut microbiota and the intestinal barrier function (82). A recent review by De Santis et al. detailed many dietary factors that may modulate the intestinal barrier (83). Here, we review some recent publications and emphasize the effects of diet-induced alterations of gut microbiota on compromising the gut barrier function. Vitamin D (VD) has been recognized as an intestinal permeability protector by inducing the expression of TJ proteins ZO-1 and claudin-1. In VD receptor (VDR)-knockout mice, more severe experimental colitis has been observed, suggesting the protective effect of VD on the mucosal barrier (84). However, another group have recently found that VDR deficiency lowers, whereas VD treatment upregulates, the expression of claudin-2, a pore-forming TJ protein, which renders the intestinal epithelium leaky (85). Further analysis confirmed that VDR enhanced claudin-2 promoter activity. The exact role of VD and VDR on modulating intestinal permeability is therefore unclear and should be investigated carefully in association with gut microbiota. In a recent study by Desai et al., a low-fiber diet consumption was found to trigger the expansion of mucus-degrading bacteria, including *Akkermansia muciniphila* and *Bacteroides caccae* (45). As a result, the thickness of mucus is significantly decreased in mice fed with fiber-deficient diets, although the transcription of *Muc2* gene was surprisingly heightened, possibly as a compensatory response. The thinner mucus and compromised intestinal barrier function lead to a higher susceptibility to certain colitis-causing pathogens (45). Moreover, a diet high in saturated fat has been shown to greatly decrease *Lactobacillus* and increase *Oscillibacter*, and these changes were correlated with significantly increased permeability in the proximal colon (86). Furthermore, studies revealed that the abundance of the *Oscillospira* genus was negatively correlated with the mRNA expression of barrier-forming TJ protein ZO-1.

### Stress-Induced Gut Leakiness

Under certain circumstances, stress-induced alterations of gut microbiota and the impaired intestinal barrier would allow the occurrence of microbial translocation. Burn injury and alcohol consumption are examples of such stress. Burn injury results in increased intestinal permeability, which is mediated by increased activity of myosin light-chain (MLC) kinase (87, 88). It is known that MLC phosphorylation or kinase activation can trigger epithelial TJ opening (89–91). In burn injury, TJ proteins, including ZO-1, occluding, and claudin-1, are redistributed, which can be reversed by adding an MLC phosphorylation inhibitor (87). In addition, both humans and mice experiencing burn injury undergo similar alterations of gut microbiota, in particular, with increases of the abundance of bacteria from the *Enterobacteriaceae* family (88). Importantly, microbial translocation of these Gram-negative aerobic bacteria has been observed. Another research group, using a different burn injury mouse model reported increased colonic permeability together with reduced aerobic and anaerobic bacterial populations in the gut microbiota, particularly those producing butyrate (92). As a consequence, the butyrate level in the stool was significantly decreased in mice with burn injury. Interestingly, when the experimental mice received fecal microbiota transplant, their altered bacterial counts and impaired mucosal barrier function were reversed, suggesting direct involvement of microbiota in causing gut leakiness after burn injury.

Chronic alcohol consumption is responsible for intestinal barrier dysfunction, alterations on both the quality and quantity of gut microbiota, LPS translocation, and alcoholic liver disease (ALD). In both human and mouse, it has been well established that alcohol can disrupt intestinal barrier function, which is closely related to increased tumor necrosis factor (TNF) production from intestinal monocytes/macrophages and enterocytes bearing TNF-receptor 1, followed by downstream activation of MLC kinase (93). Notably, when mice given chronic alcohol also received oral antibiotic treatment, to remove the microbiota, the level of TNF production and intestinal permeability decreased to levels comparable to those in control mice (93). This indicates that the alcohol-induced, TNF-mediated gut leakiness is greatly dependent on gut microbiota. Indeed, though the mechanism is unknown, alcohol administration alters microbiota qualitatively and quantitatively in both human and mouse (94). Bacterial overgrowth has been observed with alcohol consumption, whereas antibiotics can decrease the bacterial load and attenuate ALD (53, 93, 95–97). Interestingly, probiotic *Lactobacillus* is significantly suppressed during alcohol consumption (53, 97). Directly supplying *Lactobacillus* strains or indirect stimulation of *Lactobacilli* with prebiotics or diets can decrease bacterial overgrowth, restore mucosal integrity of the intestine, and suppress microbial translocation (53, 94, 98, 99). Microbial translocation, especially the translocation of LPS, is involved in ALD development and progression as evidenced by the lack of ALD in mice deficient of TLR4 (100, 101). It is worth noting that some bacteria species can produce alcohol, including *E. coli* and *Weissella confusa*, and this may be the mechanism by which they compromise the intestinal barrier function (102, 103).

Infections can play a role in regulating the mucosal barrier. A good example is *Helicobacter pylori*, a Gram-negative bacterium infecting the human stomach (104). *H. pylori* is known to directly increase epithelial permeability by redistributing TJ protein ZO-1 (105, 106). In addition, bacteriophages, which are usually not considered pathogenic to mammals, can have an impact on the leaky gut. When rats were given a bacteriophage cocktail containing phages against *Salmonella enterica*, disruption of the intestinal barrier integrity was observed (107). The authors speculated that the gut microbiota might have been affected by bacteriophages, but sequencing data were not supplied to support their claims.

Taken together, perturbation of gut microbiota, which may be the consequence of diverse interventions, can lead to increased intestinal permeability and translocation of bacterial components and products. Such microbial translocation can subsequently trigger an abnormal immune response, causing inflammation and/or tissue damage in extraintestinal organs.

## Leaky Gut and Autoimmune Disorders

Several disease states have been associated with gut microbiota dysbiosis, intestinal barrier dysfunction, and microbial translocation. These include Alzheimer’s disease, ALD, cancer, and multiple autoimmune disorders. Autoimmune disorders are characterized by the generation of autoantibodies against self-antigens that attack the body’s own tissues, resulting in damage. Genetic and environmental triggers have been long known as the major contributors to the development of autoimmunity. Increasing evidence in recent years suggests that microbial translocation and intestinal barrier dysfunction, which may be affected by gut microbiota, are another important causative element for autoimmune disorders (2–6). T1D and SLE are examples discussed below that reveal advancements in the understanding of the mechanisms behind the interaction between the leaky gut and autoimmune disorders.

### Type 1 Diabetes

Type 1 diabetes is an organ-specific autoimmune disorder characterized by an autoimmune response against the host’s own pancreatic β cells, leading to insufficient insulin production from the pancreas (108). Some argue that the leaky gut is only an outcome of disease progression rather than an initiator or exacerbator of disease (109), but this should not be the case for T1D. This is supported by the following evidences. First, studies utilizing human subjects affected by T1D or T1D-prone animal models have indicated that impaired intestinal barrier function occurs before disease onset (110–112). Second, the pathogenic role that increased intestinal permeability plays in T1D is zonulin-dependent, and the production of zonulin relies on bacterial colonization (113). Reversion of intestinal barrier dysbiosis by adding a zonulin inhibitor ameliorated T1D manifestations in disease-prone rats (114). Third, a recent study has provided evidence that microbial translocation contributes to T1D development (115). In streptozotocin-induced T1D, mice treated with streptozotocin harbor a distinct microbiota compared to vehicle-treated controls. Importantly, gut bacteria were shown to be able to translocate into pancreatic lymph nodes (PLNs) and contribute to T1D development (115). When mice were treated with oral antibiotics, PLNs appeared to be sterile and the disease was attenuated. Further analysis revealed that the translocated bacteria in PLNs triggered NOD2 activation and exacerbated T1D. Altogether, these results suggest an essential role for the leaky gut in driving the progression of T1D.

### Systemic Lupus Erythematosus

Systemic lupus erythematosus, or lupus, is an autoimmune disorder characterized by severe and persistent inflammation that leads to tissue damage in multiple organs (116). Although SLE affects both men and women, women of childbearing age are diagnosed about nine times more often than men. LPS, a cell wall component of Gram-negative bacteria, can promote SLE development and disease progression upon penetration of the intestinal epithelium and translocation into tissues (117). In SLE patients, the higher level of soluble CD14 suggests an increase in LPS, as soluble CD14 is released from monocytes when the cells are exposed to LPS (118). Activation of TLR4 exacerbates lupus development (119–121). Mice spontaneously develop lupus when TLR4 responsiveness is increased, whereas the exacerbated disease phenotype can be significantly ameliorated when the commensal gut flora is removed by antibiotic treatment (121). This clearly indicates that TLR4 hyperresponsiveness to gut flora (which contains LPS) contributes to the pathogenesis of SLE. Moreover, the development of lupus in wild-type mice (C57BL/6 or BALB/c) immunized with phospholipid-binding proteins can be facilitated by the administration of LPS (122–124). Conversely, inhibition of TLR4 results in reduced autoantibody production and lowered renal glomerular IgG deposits in lupus-prone mice (125, 126). Taken together, these data suggest that LPS stimulation and TLR4 activation as disease-initiating factors for SLE. Lipoteichoic acid (LTA), a component of the Gram-positive bacterial cell wall, can also promote lupus disease. The expression of TLR2, the receptor of LTA, has been reported to be increased in SLE patients (127). In lupus-prone mice, TLR2 activation triggers lupus nephritis, whereas TLR2 knockout attenuates lupus-like symptoms (125, 128–130). Recently, another bacterial antigen that may mimic self-antigens has been recognized to induce autoantibody production (131).

Several downstream proteins in the TLR signaling cascade are highly relevant to the pathogenesis of SLE and are potential therapeutic targets, including MyD88, IRAKs, and IFNα (132). Deficiency of MyD88, in particular, has been shown to ameliorate lupus disease in MRL/lpr mice (133, 134), suggesting a potential role for TLRs to communicate with harmful bacteria in the gut microbiota. Conversely, there is a paucity of data pertaining to members of the NLR family. The most extensively characterized NLRs are associated with inflammasome formation (135, 136). Loss of NLRP3 and AIM2 inflammasome function was found to significantly contribute to lupus pathogenesis (137). Interestingly, both of these inflammasomes were found compromised in NZB mice, a lupus-prone model. Consistent with this finding, loss of ASC (apoptosis-associated speck-like protein containing CARD), a common adaptor protein required for inflammasome formation in B6-*Fas^lpr^* mice led to exacerbation of lupus-like disease (138). These results suggest a potential role for NLRs to recognize protective bacteria in the gut microbiota. Therefore, it appears that TLRs and NLRs make distinct contributions to lupus pathogenesis by sensing harmful and protective bacteria, respectively. Both types of bacteria can come from gut microbiota through microbial translocation, especially in the presence of a leaky gut.

## Reversing the Leaky Gut as a Potential Therapy

Considering the contributions of leaky gut and bacterial translocation to inflammation and multiple diseases, reversing gut leakiness appears to be an attractive therapeutic strategy. Prebiotics and probiotics, for example, can be used to reduce intestinal permeability (139). Diverse probiotic species have been uncovered that possess the properties to protect the intestinal barrier through targeting different components of the mucosal barrier system. The human commensal *Bacteroides fragilis* may serve as such a probiotic (140). In a mouse model, autism spectrum disorder (ASD) has been shown to be accompanied by intestinal barrier dysfunction, gut microbiota dysbiosis, and leakiness of 4-ethylphenylsulfate (4EPS), which originates from the commensal bacteria. When 4EPS was given to wild-type mice, it directly caused behavioral abnormalities similar to ASD mice. Treatment with *B. fragilis* reduced the translocation of disease-causative 4EPS, and significantly ameliorated the behavior defects. The therapeutic benefit of *B. fragilis* is believed to be due to its ability to alter microbial composition and enhance intestinal barrier function (140). *B. fragilis* is also known for its capability to induce the development of Foxp3^+^ regulatory T cells, a process regulated by another product of *B. fragilis*, polysaccharide A (PSA) (141, 142). *B. fragilis* and PSA are beneficial against inflammatory diseases, such as colitis and experimental autoimmune encephalomyelitis (141, 143). The application of *B. fragilis* to prevent the leaky gut and reverse autoimmunity warrants further investigation. In a practical point of view, probiotic candidates with different targets on reversing the leaky gut may synergistically act to attenuate disease as thus may serve as a probiotic cocktail. As probiotics are generally considered safe, it is anticipated that they will become cost-effective treatment options for people with autoimmune diseases in the foreseeable future. This is a very young but exciting field in which much still remains to be learned.

## Author Contributions

All authors listed have made substantial, direct, and intellectual contribution to the work and approved it for publication.

## Conflict of Interest Statement

The authors declare that the research was conducted in the absence of any commercial or financial relationships that could be construed as a potential conflict of interest.
